# Cancer Burden in China during 1990–2019: Analysis of the Global Burden of Disease

**DOI:** 10.1155/2022/3918045

**Published:** 2022-04-14

**Authors:** Shu-Zhen Zhang, Li Zhang, Long Xie

**Affiliations:** ^1^The State Key Laboratory Breeding Base of Basic Science of Stomatology (Hubei-MOST) & Key Laboratory of Oral Biomedicine Ministry of Education, School & Hospital of Stomatology, Wuhan University, Wuhan, China; ^2^Department of Obstetrics and Gynecology, Hubei Provincial Hospital of Integrated Chinese and Western Medicine, Wuhan, China

## Abstract

This study reports the risk factors, trends, and burden of cancer in China from 1990 to 2019 from the Global Burden of Diseases. The incidence, mortality, and DALY of all cancers in China for the past 30 years were analyzed. In 2019, the age-standardized rates (ASRs) of cancer incidence, mortality, and DALY in China were 232.42/100 000, 136.72/100 000, and 3288.22/100 000, respectively. The five cancers with the highest age-standardized incidence rates were lung, stomach, colorectal, breast, and prostate cancers. From 1990 to 2019, the number of new cancer cases, deaths, and DALY increased by 168.78%, 86.89%, and 51.20%, respectively. The ASR increased by 22.21% for incidence and decreased by 19.01% and 27.19% for mortality and DALY, respectively, and their corresponding average annual percent change values were 0.71, -0.80, and -1.26, respectively. The main risk factors for cancer in China were smoking, air pollution, dietary factors, and alcohol use. From 1990 to 2019, the cancer incidence rate was on the rise, and cancer mortality and DALY rates were declining; however, these characteristics vary by cancer site. Therefore, current prevention strategies should be reoriented, and specific strategies for cancers in different sites should be established to prevent the increase in cancer cases.

## 1. Introduction

A total of 18.1 million new cancer cases worldwide and 9.6 million deaths from cancer were estimated in 2018, of which approximately 24% of new cases and 30% of deaths were attributed to China where lung cancer is the most common cause of death [1, 2]. Cancer has become an important cause of death in this country, and its disease burden has been increasing rapidly [3].

The rapid economic growth in China is accompanied by changes in population structure and the epidemic spectrum [4, 5]. The aging population, increased environmental pollution, uncontrolled chronic infections, and heightened exposure to risk factors (such as smoking, obesity, dietary patterns, and lack of physical activity) have continuously increased the burden of cancer [6, 7]. In urban areas, the cancer spectrum has shifted to Westernization as characterized by the high incidence of female breast and colorectal cancer (CRC); esophageal cancer, liver cancer, and cervical cancer are still common in rural areas [8].

Accurate and effective cancer burden information is essential for cancer prevention and control. The Global Burden of Disease Study 2019 (GBD 2019) estimated the burden of diseases, injuries, and risk factors for 204 countries and territories and selected subnational locations for the analysis of cancer incidence and mortality trends. The findings will aid in formulating correct policies, reasonable resource allocation, and effective prevention and treatment measures to reduce the burden of cancer. Average annual percentage change (AAPC) uses the natural logarithm of the regression line fitting rate and is widely used to measure the trend of age-standardized rates (ASRs) in a specific period or to evaluate the effectiveness of current prevention strategies [9].

This study employed the latest data of GBD 2019 to comprehensively analyze the risk factors, incidence, mortality, DALY, and trends of all cancers in China.

## 2. Materials and Methods

Data on the burden of cancer in China were obtained from the online data source tool Global Health Data Exchange (GHDx) query tool (http://ghdx.http://healthdata.org/gbd-results-tool), including cancer incidence, mortality, and disability-adjusted life years (DALY) rate based on gender and age in China from 1990 to 2019 and the risk factors of cancer leading to DALY in 2019.

### 2.1. Statistical Analysis

The burden of cancer was validated by calculating the ASR of incidence, mortality, and DALY with Ren's method [9]. The National Cancer Institute Joinpoint regression program software was used to calculate the AAPC of ASRs of incidence, mortality, and DALY and its statistically significant differences in the overall trend from 1990 to 2019. If the AAPC and the lower limit of the 95% confidence interval (CI) are both >0, then the ASR is exhibiting an increasing trend; if the estimated APC and the upper limit of the 95% CI are both <0, then the ASR is showing a decreasing trend; otherwise, ASR is considered stable.

## 3. Results

### 3.1. Estimated Numbers of New Cancer Cases and Incidence

Table 1 shows the estimated number of new cases, age-standardized incidence rates (ASIRs), and changes in 28 types of cancers in China in 1990 and 2019. In 2019, the estimated number of new cancer cases was 4,551,170, which comprised of 58% men and 42% women and was equivalent to 13,000 new cancer cases every day. From 1990 to 2019, the number of new cases increased by 166.73% in men and 151.81% in women; however, the corresponding ASIR increased by 26.17% to 294.59 per 100 000 for men and 27.21% to 181.72 per 100,000 for women. Overall, the number of new cases of all cancer types increased by 168.78%, and the ASIR increased by 22.21%.

For both sexes, the five sites susceptible to cancer were lung, stomach, colorectal, breast, and non-melanoma skin with ASIR of 41.71/100 000, 30.64/100 000, 30.55/100 000, 18.32/100 000, and 14.32/100 000, respectively, in 2019. The five highest ASIRs of cancer in 2019 were lung cancer, stomach cancer (SC), CRC, esophageal cancer, and prostate cancer in males and breast cancer, lung cancer, CRC, stomach cancer, and non-melanoma skin cancer in females. Compared with that in 1990, the ASIR of stomach cancer (SC) in both sexes in 2019 decreased by 18.44%; male esophageal cancer and SC decreased by 23.55% and 7.28%, respectively; and female SC decreased by 38.20%.

**Figure 1:**
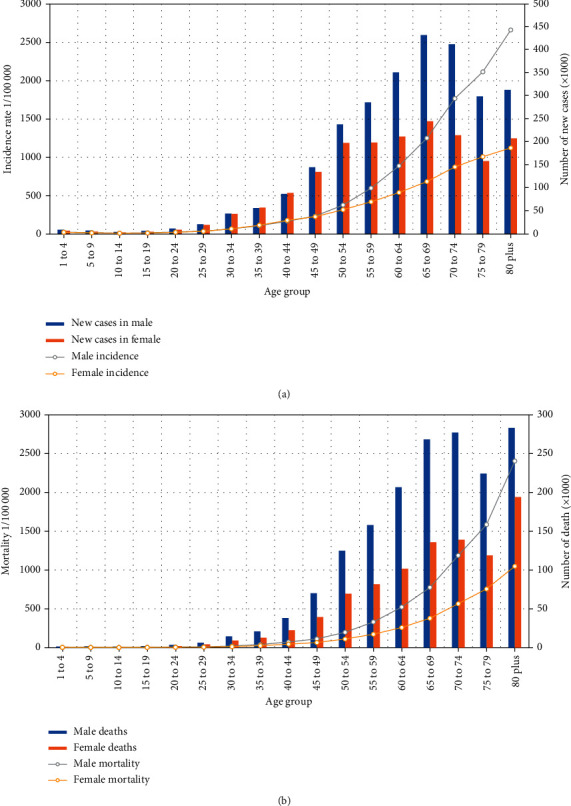
Trend of cancer by crude incidence rate, mortality rate, and DALY in China, 1990–2019.

Table 2 and Figure 1(a) show that the incidence of cancer increased with age. In 2019, the ASIR was the highest in males and females aged 80 years at 2 405.36 (1972.62, 2 827.94) per 100 000 and 1049.41 (804.53, 1 265.21) per 100 000, respectively. The 65–69 age group had the highest number of new cases at 2 405 360 for males and 1 049 410 for females.

### 3.2. Estimated Numbers of Death and Mortality

Table 3 shows the estimated number of deaths, the age-standardized mortality rate (ASMR), and changes in 28 types of cancers in China from 1990 to 2019. In 2019, the estimated number of deaths was 2 640 200, 65% of which was attributed to men and 35% to women. The number of deaths increased by 96.14% in males and 72.11% in females, but their corresponding ASMRs decreased by 12.79% to 192.28 per 100 000 and 27.21% to 92.65 per 100 000, respectively. Generally, the number of deaths of all cancer increased by 86.89%, and the ASMR decreased by 19.01%.

In both sexes, the five cancers with the highest mortality in 2019 were lung, stomach, colorectal, esophageal, and liver cancer with ASMR of 38.70/100 000, 21.72/100 000, 13.86/100 000, 13.15/100 000, and 9.41/100 000, respectively, in 2019. The top five ASMR of cancer in 2019 were lung cancer, stomach cancer, esophageal cancer, CRC, and liver cancer in males and lung cancer, stomach cancer, CRC, breast cancer, and esophageal cancer in females.

Compared with that in 1990, deaths from liver cancer in 2019 decreased by 19.25%, and the ASMR of stomach cancer, esophageal cancer, and liver cancer decreased by 42.43%, 40.44%, and 63.79%, respectively, for both sexes. Compared with that in 1990, the ASMR of SC, esophageal cancer, and liver cancer in males in 2019 decreased by 35.48%, 28.96%, and 60.27%, respectively, and the ASMR of SC, breast cancer, and esophageal cancer in females decreased by 53.38%, 1.53%, and 59.10%, respectively.

Table 2 and Figure 1(b) indicate the estimated age-specific cancer deaths and age-specific death rates in 2019. The mortality of cancer increased with age and peaked in the over 80 age group with up to 2662.53 per 100 000 in males and 1124.52 per 100 000 in females. The estimated number of deaths increased with age and peaked in the 80 plus age group for both sexes. In all age groups, male mortality rates and deaths were consistently higher than those for females.

### 3.3. Estimated Numbers of DALY and Age-Standardized DALY

Table 4 shows the estimated number of DALYs, the age-standardized DALY rate, and changes in 28 types of cancers in China from 1990 to 2019. From 1990 to 2019, the DALY increased by 58.14% in men and 39.55% in women; however, the corresponding age-standardized DALY decreased by 22.46% from 5642.34 per 100 000 person-years in 1990 to 4375.35 per 100 000 person-years in 2019 for males and by 33.43% from 3456.54 per 100 000 person-years in 1990 to 2301.10 per 100 000 person-years in 2019 for females. Generally, the number of DALY of all cancer increased by 51.20%, and the age-standardized DALY rate decreased by 27.19%.

The five cancers with the highest DALY were lung, stomach, esophageal cancers, CRC, and liver cancer in males and lung, breast, stomach, colorectal, and cervical cancers in females. In terms of age-standardized DALY, the three cancers with the highest increase were pancreatic cancer (67.61% increase; 81.48 to 136.57 per 100 000 person-years), kidney cancer (58.78% increase; 21.59 to 34.28 per 100 000 person-years), and female ovarian cancer (44.90% increase; 55.57 to 80.52 per 100 000 person-years). The three cancers with the highest decrease were Hodgkin lymphoma (73.33% decrease; 18.56 to 4.95 per 100 000 person-years), liver cancer (65.63% decrease; 769.11 to 264.31 per 100 000 person-years), and leukemia (50.92% decrease; 333.77 to 163.82).

### 3.4. Risk Factors

**Figure 2:**
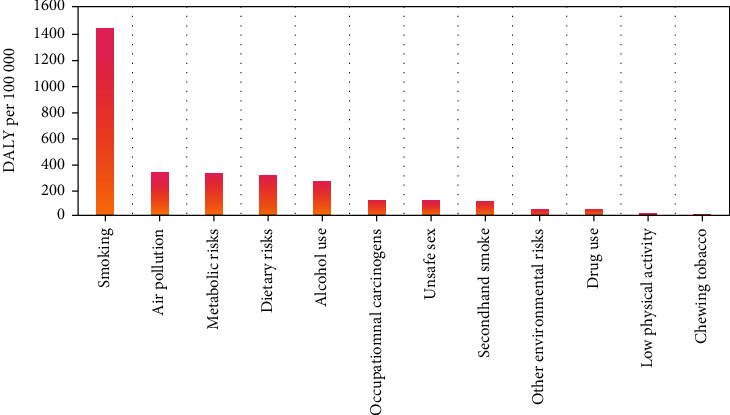
Risk factors of cancer contributing to DALYs in China, 2019.

Tobacco was the leading risk factor associated with the highest DALY rates in 2019 with the value of 1541.09 (1142.75–1995.33) per 100 000 person-years, including chewing tobacco at 3.77 (1.94–6.25) per 100 000 person-years, smoking at 1434.31 (1082.14–1832.24) per 100 000 person-years, and secondhand smoking at 103.01 (58.66–156.85) per 100 000 person-years. Other risk factors for DALY rates were air pollution at 330.92 (241.17–436.40) per 100 000 person-years, metabolic risks at 321.41 (100.90–662.33) per 100 000 person-years, dietary risks at 303.84 (136.96–590.18) per 100 000 person-years, alcohol use at 261.66 (182.29–358.65) per 100 000 person-years, and occupational carcinogens at 115.27 (78.47–159.21) per 100 000 person-years (Figure 2).

### 3.5. Trends in Cancer ASRs of Incidence, Mortality, and DALY

Table 5 shows temporal trends in cancer incidence, mortality, and DALYs by sex for all cancer in China from 1990 to 2019. For all cancer types, the ASIR showed increasing trends for both sexes, and the ASMR and age-standardized DALYs exhibited decreasing trends.

Among all cancers in males, the ASIR of most cancers showed an upward trend; leukemia, esophageal cancer, and liver cancer showed a downward trend; and stomach cancer and Hodgkin lymphoma remained stable. From 1990 to 2019, the ASMR showed an upward trend for 11 cancers and a downward trend for 14 cancers. The age-standardized DALY rate showed an upward trend for 12 cancers and a downward trend for 13 cancers (Table 5).

Among all cancers in females, the ASIR of most cancers showed an upward trend and bladder cancer, lip and oral cavity cancer, and mesothelioma; Hodgkin lymphoma, leukemia, stomach cancer, esophageal cancer, and liver cancer showed a downward trend; and larynx cancer remained stable. From 1990 to 2019, the ASMR showed an upward trend for 7 cancers and a downward trend for 17 cancers. Only cervical cancer and non-Hodgkin lymphoma remained stable. The age-standardized DALY rate showed an upward trend for 4 cancers and a downward trend for 18 cancers. Four cancers remained stable (Table 5).

## 4. Discussion

By analyzing data from the GBD database, this study estimated the incidence, mortality, and DALY of 28 cancers in China from 1990 to 2019; assessed the corresponding incidence, mortality, and DALY trends of each cancer; and examined the risk factors related to the DALY of cancer. The results can help government decision-makers understand the burdens and trends of various types of cancer in this country and aid in formulating and implementing effective control measures and rational allocation of resources.

From 1990 to 2019, the ASIR of all cancers in China showed a steady increase, whereas ASMR and age-standardized DALY exhibited a downward trend. However, these results vary depending on the cancer site. Although the cases of cancer are the highest among males and females in the 65–69 age group, the age-specific incidence rate and age-specific death rate are the highest among the aged 80 plus group. Aging significantly affects normal cells in the cancer microenvironment and promotes cancer progression and metastasis [10].

### 4.1. Lung Cancer

Lung cancer is the leading cause of death in China. Males have the highest incidence of lung cancer. The continuous increase in the incidence of lung cancer from 1990 to 2019 is related to the popularization of advanced diagnostic equipment. From 2005 to 2014, the use rate of chest radiography decreased from 50.2% to 31.0%, and that of chest CT increased from 65.8% to 81.4% [11].

Smoking is the main determinant factor leading to lung cancer. China is currently the largest tobacco production and consumer in the world [12]. More than 50% of lung cancer can be attributed to smoking [13]. Passive inhalation of secondhand smoke in work or living environment is also related to the occurrence of female lung cancer [14]. Among nonsmokers in China, approximately 16% of lung cancer cases may be attributable to passive smoking [15]. Quitting this activity reduces the risk of lung cancer and death by 50% [16]. Rapid industrialization has increased industrial waste gas emissions and environmental-related diseases and reduced air quality [17]. According to the report of the Ministry of Ecology and Environment of China in 2019, the air quality has reached a dangerous level in 180/337 (53.4%) cities [18]. Polluted air has aggravated the incidence of lung cancer. Smoking and polluted air synergistically increase the DALY in China.

The high and continuously increasing ASMR of lung cancer is related to the following factors. Firstly, the increasing incidence of lung cancer has led to an increasing trend of mortality. The second is delayed treatment, with approximately two-thirds of patients losing the best opportunity for surgery [19]. Finally, the main type of lung cancer has changed from squamous cell carcinoma to adenocarcinoma [11].

### 4.2. Stomach Cancer

Although the incidence and mortality of SC are decreasing, the large number of cases and deaths in China still represents a huge burden. SC has the second-highest morbidity and mortality rate in China after lung cancer. For the past 30 years, the ASR of incidence, mortality, and DALY of SC showed a decreasing trend.

SC is linked to smoking and the heavy use of pickled fish and vegetables and *Helicobacter pylori* infection [20]. A history of smoking for more than 30 years is related to a poor prognosis in patients undergoing gastrectomy [21]. The following factors can explain the decreasing trend of SC incidence and prevalence. Firstly, the overall current smoking rate has decreased [13]. Secondly, substantial progress was made in the control of *H. pylori* infection. Its prevalence has decreased from 1983 to 2018. The overall prevalence rates were 63.8% in 1983–1994, 57.5% in 1995–2005, and 46.7% in 2006–2018 [22]. Thirdly, the rapid development of China's economy has accelerated this trend. Everyone pays attention to health awareness, and clean tap water has become easy to obtain. The diet has become healthy, and the nutritional status of the Chinese people has improved significantly. These factors can explain the reduced incidence of SC.

The mortality rate of SC continuously decreased from 1990 to 2019. The SC mortality rate is on the decline because of the gradual increase in SC screening tests. Early treatment, in-depth understanding of SC, and technological development may contribute to the reduction in SC mortality [23, 24]. Despite the decline in SC mortality in China, the 5-year survival rate was still low compared with that in other developed countries. The overall 5-year survival rate for SC is 46% in China, 58% in the United States, 64.6% in Japan, and 76.5% in South Korea [25–27]. Policymakers should focus on the following areas. Firstly, financial resources must be directed to barium fluorescence photography or endoscopy for high-risk groups (i.e., people who test positive for *Helicobacter pylori*). Secondly, public health education helps improve the early detection of SC [28]. Finally, the government should strengthen tobacco control, raise the tobacco tax rate, and reduce the number of smokers.

### 4.3. CRC

In China, CRC was the fifth most common cause of cancer and cancer death in 2014 [3]. According to the GBD2019 database, CRC has become the third leading cause of morbidity and mortality in 2019. From 1990 to 2019, its ASIR increased by an average of 3.66% per year, and ASMR increased by an average of 1.39% per year (Table 5). Dietary patterns were the most important risk factor for DALY. Previous researches reported that the main risk factor for CRC is low vegetable intake with a PAF of 17.9%. Approximately 8.9% of CRC incidence and mortality is attributed to physical inactivity. Other factors, such as high red and processed meat intake, low fruit intake, alcohol drinking, overweight/obesity, and smoking, account for 8.6%, 6.4%, 5.4%, 5.3%, and 4.9% of CRC cases, respectively [29].

The increasing incidence is associated with the following causes: (1) accelerated industrialization; (2) rapid economic development; (3) eating habits shifted to Western diet; (4) increased obesity, smoking, and drinking; and (5) increased aging population [30].

CRC is asymptomatic in the early stage, and symptoms may not appear until the late stage. Colonoscopy screening is an effective way to prevent and detect early cancer and improve survival and prognosis [31] and can greatly reduce the occurrence of colon cancer by allowing the early removal of intestinal polyps that may develop into CRC [30]. However, the Chinese population lacks awareness of CRC, and their acceptance of colorectal endoscopy screening is extremely low [32]. The average time of prehospital delay for Chinese patients with CRC is 18 weeks, suggesting a delay in diagnosis and treatment [33]. CRC is most common in people aged 60–74 years, and older people over 74 years old are highly likely to die from CRC [34]. Given that China has entered an aging society, the percentage of individuals over 65 years old is expected to reach 18% in 2030 [35]. The increase in mortality rate is related to inadequate screening, delayed diagnosis, and accelerated aging.

Given the low acceptance of colonoscopy in the Chinese population, large-scale use of colonoscopy for screening may not be the best method [36]. The limited medical expenses and medical staff and equipment and the huge population were also factors to consider. Fecal immunology test may be suitable for China because of its convenience, economy, and effectiveness [34]. Participants with positive fecal occult blood tests are more likely to undergo colonoscopy than those with negative fecal occult blood tests [36]. Stool screening is used to identify high-risk patients with CRC, and colorectal endoscopy is an economical method that will undoubtedly increase the detection rate.

### 4.4. Breast Cancer and Cervical Cancer

Breast cancer is the most common cancer, and cervical cancer is the sixth most common among females in China in 2019 according to GBD 2019. During the study period, the trend of ASIRs of breast cancer and cervical cancer in women was on the rise. Particularly, the ASRs of mortality and DALY of breast cancer in women were high but decreasing, and those of mortality and DALY of cervical cancer in women remained stable.

With China entering an aging society and shifting its dietary habits and lifestyle to Western cuisine, increased age and obesity lead to a significant increase in breast cancer mortality and DALY [37]. From 2005 to 2015, the attributable fraction for breast cancer (postmenopausal) increased from 8.8% to 11.9% due to the increase in overweight and obesity cases in urban females [38]. According to a dose-response meta-analysis from prospective studies, 1 h/day increment of sedentary behavior or TV viewing daily can increase the risk of breast cancer by 1% or 2%, respectively. The downtrend in ASRs of mortality and DALY of breast cancer may be related to early detection.

Long-term human papillomavirus (HPV) infection leads to cervical cancer, the primary factor causing the burden of cervical cancer [39]. The overall high-risk HPV prevalence was 17.7% in China [40], and this positive rate is directly related to the severity of cervical lesions [41]. Some developed countries have successfully prevented cervical cancer through screening, and the promotion of the HPV vaccine will further affect cervical cancer [39]. The increase in cervical cancer incidence and mortality may be related to insufficient screening coverage, limited HPV vaccine application, and high cost of HPV vaccines. From 2016 to 2018, three imported HPV vaccines were approved in China, but the high price of imported vaccines hindered their promotion [42]. At the end of 2019, the domestic bivalent HPV vaccine was approved, and the price was cheaper than that of the imported HPV vaccine. This decision will aid in promoting the HPV vaccine and preventing cervical cancer in the future.

### 4.5. Other Cancers

The prevention and control of some cancers have achieved remarkable results. In this study, the ASRs of incidence, mortality, and DALY of liver cancer, esophageal cancer, leukemia, and Hodgkin lymphoma in both sexes exhibited a decreasing trend. The remarkable results in the prevention of liver cancer in China can be attributed to the routine HBV immunization of infants implemented since 1992 [43]. The effectiveness of esophageal cancer prevention can be attributed to improved sanitation, preservation of food using cold storage, and consumption of less pickled but nutritious foods, such as fruits and vegetables [43]. Leukemia is the main category of childhood tumors. The reduction of its burden is due to the following factors: (1) One-child policy allows each family to pay attention to the health of children; (2) advancement of early diagnosis technology for leukemia provides effective help for the early detection of leukemia; and (3) progress on the treatment of leukemia [44]. Hodgkin lymphoma has an extremely low incidence (0.57 per 100 000) in mainland China. Owing to medicinal development, the 5-year overall survival rate of Hodgkin lymphoma can reach up to 97.7% [45]. All the above factors contribute to the reduction in the cancer burden of Hodgkin lymphoma in China.

For pancreatic cancer, non-Hodgkin lymphoma, male oral cancer, multiple myeloma, gallbladder, and biliary tract cancer, the ASRs of incidence, mortality, and DALY were all on the rise from 1990 to 2019. Therefore, the previous prevention and control strategies are not in line with the actual status.

### 4.6. Recommendations

The overall cancer burden showed a downward trend from 1990 to 2019. However, the cancer burden remains heavy due to the large population in China. The top three risk factors that cause the burden of cancer DALY in 2019 were smoking, air pollution, and metabolic risk. For smoking, implementing an active tobacco control plan is urgent. Increasing the price of tobacco by placing a tobacco excise tax can reduce the number of smokers [46]. Many cities have implemented anti-smoking laws, but law enforcement must be strengthened. Tobacco control mass media promotion helps reduce smoking prevalence [47]. From 2013 to 2017, the air pollution indicators of 74 key cities across the country decreased significantly but still exceeded China's Grade II criteria ambient air quality standards [48]. The government should improve the energy supply structure, increase the proportion of clean energy supply, and promote new energy vehicles to reduce polluting exhaust emissions. With the economy development, the Chinese lifestyle has gradually transitioned to Western dietary habits that increased the metabolic risk factors, such as hypertension, hyperlipidemia, hyperglycemia, and high body mass index. Following the Chinese dietary guidelines can reduce overall mortality [49].

This study has important limitations. Firstly, the reliability of this research depends on the quality and quantity of available data sources. GBD data estimation is largely derived from the choice of covariates in the model and regional model. Secondly, cancer mortality estimates are mainly based on vital registration data, cancer registration data, and other data sources. Lastly, the assessment of cancer burden in this study is limited to standard epidemiological parameters; hence, the economic burden of the disease is ignored. These limitations may indicate underestimation of the actual burden of cancer.

Current prevention strategies should be reoriented, and specific strategies for cancers in different sites should be established to prevent the increase in cancer cases.

## Figures and Tables

**Table 1:** Total all-age incidence and age-standardized incidence rates for different types of cancer and their percentage change by gender in China, 1990–2019.

Morphology	Gender	Total all-age incidence, no. ∗ 10^3^(95% UI)			Age-standardized incidence rate (95% UI), per 100,000		
		1990	2019	Change, %	1990	2019	Change, %
All neoplasms	Male	989.39 (771.24, 1204.75)	2639.01 (2074.05, 3272.87)	166.73	233.48 (185.78, 281.18)	294.59 (233.01, 362.05)	26.17
	Female	703.89 (547.24, 879.33)	1816.78 (650.32, 2265.26)	158.11	154.51 (121.09, 191.46)	181.72 (140.07, 226.34)	27.21
	Both	1693.28 (1379.69, 2013.50)	4551.17 (3716.31, 5453.52)	168.78	190.18 (156.49, 224.19)	232.42 (190.76, 277.19)	22.21

Tracheal, bronchus, and lung cancer	Male	178.99 (145.48,213.93)	576.19 (451.4,709.27)	221.91	44.29 (36.72,52.43)	61.74 (48.93,75.20)	39.40
	Female	78.05 (65.13,91.71)	256.74 (205.68,314.16)	228.94	18.01 (15.12,21.04)	24.76 (19.89,30.26)	37.48
	Both	257.04 (221.29,293.65)	832.92 (700.29,981.63)	224.04	30.20 (26.20,34.26)	41.71 (35.22,48.80)	38.11

Stomach cancer	Male	207.53 (173.24,245.61)	451.33 (357.18,560.15)	117.48	51.07 (42.99,59.92)	47.35 (38,57.95)	-7.28
	Female	109.81 (92.09,127.87)	161.49 (130.60,198.34)	47.06	25.57 (21.49,29.61)	15.80 (12.80,19.38)	-38.21
	Both	317.34 (277.90,359.32)	612.82 (513.00,728.89)	93.11	37.56 (33.08,42.27)	30.64 (25.82,36.15)	-18.42

Colorectal cancer	Male	56.86 (47.48,66.81)	390.2 (310.20,484.11)	586.25	14.16 (12.03,16.43)	41.43 (33.41,50.87)	192.58
	Female	49.05 (40.80,57.12)	217.7 (175.11,268.36)	343.83	11.30 (9.47,13.12)	21.10 (17.00,26.04)	86.73
	Both	105.91 (93.81,119.02)	607.9 (521.80,708.42)	473.98	12.52 (11.15,14.03)	30.55 (26.37,35.50)	144.01

Breast cancer	Male	0.55 (0.45,0.66)	7.11 (5.34,9.08)	1192.73	0.13 (0.10,0.15)	0.69 (0.53,0.88)	430.77
	Female	81.07 (66.34,96.52)	368.37 (290.09,463.34)	354.39	17.07 (14.02,20.30)	35.61 (28.07,44.81)	108.61
	Both	81.62 (66.87,97.10)	375.48 (296.63,469.98)	360.03	8.54 (7.07,10.11)	18.32 (14.5,22.93)	114.52

Prostate cancer	Male	26.44 (20.10,31.92)	153.45 (118.4,204.94)	480.37	8.88 (7.14,10.86)	17.34 (13.62,22.72)	95.27

Non-melanoma skin cancer	Male	20.87 (17.61,24.97)	157.05 (135.07,179.13)	652.52	5.19 (4.45,6.05)	16.35 (14.28,18.6)	215.03
	Female	18.70 (15.61,22.43)	130.89 (111.88,150.19)	599.95	4.29 (3.62,5.04)	12.72 (10.94,14.51)	196.50
	Both	39.57 (33.25,47.49)	287.94 (246.82,328.67)	627.67	4.68 (3.96,5.48)	14.32 (12.36,16.31)	205.98

Esophageal cancer	Male	115.47 (75.12,140.39)	207.92 (156.07,258.29)	80.06	28.7 (19.23,34.41)	21.94 (16.40,27.03)	-23.55
	Female	58.21 (32.17,69.77)	70.20 (46.04,88.57)	20.60	13.94 (7.82,16.58)	6.83 (4.45,8.61)	-51.00
	Both	173.69 (112.11,203.39)	278.12 (213.51,331.60)	60.12	20.97 (13.62,24.33)	13.9 (10.7,16.52)	-33.71

Cervical cancer	Female	40.68 (30.92,73.18)	109.76 (58.19,141.54)	169.81	8.41 (6.44,15)	11.01 (5.87,14.22)	30.92

Leukemia	Male	72.91 (46.41,93.79)	85.26 (64.06,105.89)	16.94	12.70 (8.33,16.31)	11.42 (8.52,14.03)	-10.08
	Female	69.81 (49.27,88.63)	69.39 (52.7,86.51)	-0.60	12.90 (9.18,16.32)	9.59 (7.34,11.75)	-25.66
	Both	142.72 (102.38,172.53)	154.65 (127.21,181.08)	8.36	12.71 (9.22,15.31)	10.47 (8.70,12.34)	-17.62

Liver cancer	Male	170.12 (137.51,206.72)	159.79 (127.26,198.81)	-6.07	36.44 (29.58,44.21)	16.36 (13.14,20.22)	-55.10
	Female	66.71 (54.24,82.01)	50.68 (40.88,62.35)	-24.03	14.97 (12.25,18.32)	4.94 (3.99,6.06)	-67.00
	Both	236.82 (199.32,280.12)	210.46 (174.83,251.2)	-11.13	25.71 (21.73,30.35)	10.46 (8.74,12.42)	-59.32

Uterine cancer	Female	24.28 (18.18,29.96)	66.74 (51.38,92.05)	174.88	5.13 (3.89,6.29)	6.39 (4.89,8.74)	24.56

Pancreatic cancer	Male	15.83 (12.77,19.08)	69.64 (55.22,86.08)	339.92	3.89 (3.19,4.64)	7.43 (5.96,9.09)	91.00
	Female	10.94 (9.21,12.67)	45.33 (36.45,55.80)	314.35	2.55 (2.16,2.96)	4.36 (3.51,5.37)	70.98
	Both	26.77 (23.13,30.26)	114.96 (98.05,133.71)	329.44	3.17 (2.77,3.57)	5.78 (4.94,6.69)	82.33

Brain and central nervous system cancer	Male	25.09 (16.49,37.77)	47.07 (30.53,61.74)	87.60	4.84 (3.21,7.23)	5.64 (3.61,7.33)	16.53
	Female	20.76 (13.75,28.16)	47.62 (34.83,62.14)	129.38	4.09 (2.75,5.55)	5.83 (4.30,7.77)	42.54
	Both	45.85 (35.18,61.35)	94.69 (73.40,114.09)	106.52	4.45 (3.47,5.94)	5.69 (4.36,6.78)	27.87

Nasopharynx cancer	Male	20.88 (17.07,24.83)	82.91 (64.82,103.69)	297.08	4.32 (3.55,5.10)	8.55 (6.75,10.65)	97.92
	Female	11.18 (8.92,13.37)	27.53 (21.33,35.04)	146.24	2.33 (1.88,2.77)	2.83 (2.19,3.59)	21.46
	Both	32.06 (27.39,36.56)	110.43 (90.34,132.40)	244.45	3.31 (2.84,3.76)	5.65 (4.67,6.75)	70.69

Bladder cancer	Male	18.36 (15.42,21.45)	82.68 (66.13,100.84)	350.33	5.46 (4.67,6.26)	9.38 (7.63,11.30)	71.79
	Female	7.19 (5.92,8.61)	17.34 (13.6,21.66)	141.17	1.75 (1.45,2.09)	1.72 (1.35,2.14)	-1.71
	Both	25.55 (22.27,29.01)	100.02 (83.24,118.65)	291.47	3.30 (2.89,3.72)	5.16 (4.33,6.10)	56.36

Non-Hodgkin lymphoma	Male	11.72 (9.47,14.57)	60.6 (47.98,76.02)	417.06	2.40 (1.97,2.93)	6.77 (5.44,8.35)	182.08
	Female	8.36 (6.78,10.31)	31.35 (25.14,38.73)	275.00	1.71 (1.41,2.06)	3.36 (2.72,4.12)	96.49
	Both	20.08 (17.05,24.08)	91.95 (76.98,108.97)	357.92	2.04 (1.76,2.40)	4.99 (4.24,5.87)	144.61

Ovarian cancer	Female	12.68 (9.91,17.51)	45.48 (33.11,57.38)	258.68	2.56 (2.00,3.58)	4.54 (3.33,5.71)	77.34

Kidney cancer	Male	6.16 (5.06,7.48)	42.55 (33.14,53.17)	590.75	1.32 (1.09,1.60)	4.63 (3.67,5.73)	250.76
	Female	4.92 (4.24,5.68)	17.27 (13.84,21.30)	251.02	1.02 (0.88,1.17)	1.92 (1.58,2.32)	88.24
	Both	11.07 (9.79,12.59)	59.83 (49.51,71.24)	440.47	1.16 (1.02,1.31)	3.21 (2.7,3.79)	176.72

Testicular cancer	Male	3.23 (2.56,4.40)	17.17 (13.72,21.57)	431.58	0.56 (0.45,0.75)	2.39 (1.90,3.00)	326.79

Lip and oral cavity cancer	Male	7.06 (5.74,8.42)	33.48 (26.15,42.01)	374.22	1.68 (1.38,1.99)	3.48 (2.75,4.31)	107.14
	Female	5.33 (4.43,6.30)	11.74 (9.47,14.48)	120.26	1.17 (0.98,1.37)	1.16 (0.94,1.43)	-0.85
	Both	12.39 (10.87,14.06)	45.22 (37.69,54.18)	264.97	1.40 (1.23,1.57)	2.25 (1.89,2.68)	60.71

Larynx cancer	Male	11.03 (8.97,13.07)	38.90 (30.45,48.73)	252.67	2.68 (2.21,3.14)	3.89 (3.07,4.84)	45.15
	Female	3.05 (2.56,3.57)	6.45 (5.13,7.88)	111.48	0.70 (0.59,0.82)	0.62 (0.49,0.75)	-11.43
	Both	14.09 (11.94,16.17)	45.35 (36.75,55.36)	221.86	1.63 (1.40,1.87)	2.18 (1.78,2.65)	33.74

Thyroid cancer	Male	2.51 (2.04,3.10)	16.11 (12.08,20.19)	541.83	0.55 (0.45,0.67)	1.74 (1.32,2.16)	216.36
	Female	7.53 (5.91,9.31)	22.97 (17.46,30.16)	205.05	1.50 (1.19,1.86)	2.41 (1.83,3.17)	60.67
	Both	10.03 (8.4,11.91)	39.08 (32.28,47.66)	289.63	1.01 (0.86,1.21)	2.05 (1.70,2.50)	102.97

Gallbladder and biliary tract cancer	Male	5.66 (4.40,9.48)	19.76 (13.24,24.91)	249.12	1.56 (1.25,2.51)	2.25 (1.52,2.79)	44.23
	Female	6.77 (5.29,11.59)	18.87 (11.32,24.68)	178.73	1.64 (1.27,2.82)	1.84 (1.10,2.41)	12.20
	Both	12.44 (10.31,20.01)	38.63 (27.35,46.51)	210.53	1.58 (1.32,2.51)	2.01 (1.41,2.41)	27.22

Multiple myeloma	Male	3.04 (2.31,4.48)	11.20 (7.47,14.92)	268.42	0.72 (0.56,1.07)	1.16 (0.77,1.54)	61.11
	Female	3.03 (2.31,4.59)	7.59 (5.20,9.87)	150.50	0.69 (0.53,1.03)	0.72 (0.49,0.94)	4.35
	Both	6.07 (4.97,8.56)	18.79 (13.68,23.33)	209.56	0.70 (0.58,0.98)	0.93 (0.67,1.15)	32.86

Malignant skin melanoma	Male	1.94 (1.14,2.62)	8.41 (4.74,11.80)	333.51	0.42 (0.26,0.57)	0.93 (0.54,1.30)	121.43
	Female	1.77 (1.09,2.85)	8.53 (4.44,12.01)	381.92	0.38 (0.24,0.61)	0.91 (0.48,1.28)	139.47
	Both	3.71 (2.57,5.21)	16.94 (10.46,21.52)	356.60	0.40 (0.28,0.56)	0.92 (0.58,1.16)	130.00

Hodgkin lymphoma	Male	4.12 (1.94,5.49)	6.22 (4.01,8.02)	50.97	0.80 (0.38,1.07)	0.78 (0.50,0.98)	-2.50
	Female	2.52 (1.07,3.57)	3.24 (2.17,4.30)	28.57	0.50 (0.21,0.70)	0.40 (0.27,0.53)	-20.00
	Both	6.64 (3.48,8.67)	9.47 (7.08,11.48)	42.62	0.65 (0.34,0.84)	0.57 (0.43,0.69)	-12.31

Other pharynx cancer	Male	2.46 (2.03,2.93)	7.68 (6.00,9.63)	212.20	0.59 (0.49,0.70)	0.77 (0.61,0.96)	30.51
	Female	0.85 (0.71,1.00)	2.41 (1.89,3.03)	183.53	0.19 (0.16,0.22)	0.24 (0.18,0.29)	26.32
	Both	3.31 (2.83,3.80)	10.09 (8.29,12.12)	204.83	0.38 (0.33,0.43)	0.49 (0.41,0.59)	28.95

Mesothelioma	Male	0.56 (0.43,0.80)	1.71 (1.32,2.16)	205.36	0.13 (0.10,0.18)	0.18 (0.14,0.22)	38.46
	Female	0.64 (0.41,1.04)	1.10 (0.7,1.41)	71.88	0.14 (0.09,0.23)	0.11 (0.07,0.14)	-21.43
	Both	1.19 (0.91,1.67)	2.81 (2.31,3.35)	136.13	0.13 (0.10,0.19)	0.14 (0.12,0.17)	7.69

**Table 2:** Age-specific number of new cancer cases, deaths, incidence rate, and mortality rates of cancer by gender in China, 2019.

Morphology	Gender	Total deaths, no. ∗ 10^3^ (95%UI)	Age-specific death rates, number in 100,000 (95%UI)	Total new cases, no. ∗ 10^3^ (95%UI)	Age-specific incidence rates, number in 100,000 (95%UI)
1 to 4	Male	1.49 (0.97, 2.28)	4.16 (2.71, 6.39)	9.53 (6.09, 14.92)	26.66 (17.03, 41.72)
	Female	1.23 (0.88, 1.65)	4.00 (2.87, 5.37)	7.39 (5.08, 10.24)	24.10 (16.57, 33.40)
	Both	2.72 (1.98, 3.63)	4.09 (2.99, 5.46)	16.92 (12.01, 23.61)	25.48 (18.09, 35.54)

5 to 9	Male	1.80 (1.25, 2.36)	4.59 (3.18, 6.02)	7.86 (5.45, 10.28)	20.01 (13.88, 26.18)
	Female	1.06 (0.81, 1.32)	3.17 (2.43, 3.96)	5.53 (4.18, 7.02)	16.58 (12.54, 21.04)
	Both	2.86 (2.20, 3.51)	3.94 (3.03, 4.83)	13.39 (10.54, 16.55)	18.43 (14.52, 22.78)

10 to 14	Male	1.37(1.02,1.83)	3.58 (2.65, 4.78)	4.74 (3.53, 6.33)	12.36 (9.20, 16.51)
	Female	0.90 (0.70, 1.11)	2.77 (2.17, 3.43)	3.79 (2.92, 4.80)	11.73 (9.02, 14.84)
	Both	2.27 (1.86, 2.76)	3.21 (2.63,3.91)	8.53(6.95,10.46)	12.07(9.83,14.80)

15 to 19	Male	2.11 (1.47, 2.72)	5.25 (3.67, 6.77)	7.19 (5.05, 9.44)	17.94 (12.58, 23.54)
	Female	1.27 (0.97, 1.56)	3.61 (2.77, 4.44)	5.05 (3.77, 6.35)	14.41 (10.75, 18.11)
	Both	3.37 (2.66, 4.04)	4.49 (3.55, 5.37)	12.24 (9.58, 14.98)	16.29 (12.75, 19.94)

20 to 24	Male	3.52 (2.50, 4.54)	8.23 (5.84, 10.61)	12.07 (8.47, 16.02)	28.23 (19.81, 37.47)
	Female	2.15 (1.53, 2.81)	5.50 (3.92, 7.20)	9.50 (6.54, 12.91)	24.30 (16.74, 33.02)
	Both	5.67 (4.40, 6.91)	6.93 (5.38, 8.44)	21.57 (16.17, 27.44)	26.35 (19.75, 33.51)

25 to 29	Male	6.40 (4.79, 7.88)	11.36 (8.50, 13.99)	21.56 (15.86, 27.64)	38.26 (28.15, 49.04)
	Female	4.14 (2.91, 5.52)	7.62 (5.36, 10.16)	19.97 (13.43, 27.47)	36.73 (24.71, 50.54)
	Both	10.54 (8.33, 12.70)	9.52 (7.52, 11.47)	41.53 (31.31, 52.61)	37.51 (28.28, 47.52)

30 to 34	Male	14.58 (11.32, 17.86)	22.33 (17.33, 27.35)	44.76 (34.09, 56.36)	68.55 (52.21, 86.31)
	Female	9.13 (6.50, 11.99)	14.32 (10.20, 18.79)	44.07 (30.53, 58.90)	69.07 (47.86, 92.32)
	Both	23.72 (19.07, 28.49)	18.37 (14.77, 22.07)	88.83 (68.21, 110.84)	68.81 (52.83, 85.86)

35 to 39	Male	20.90 (16.07, 26.16)	40.66 (31.26, 50.86)	56.59 (42.80, 71.81)	110.10 (83.28, 139.71)
	Female	12.90 (9.30, 16.72)	26.05 (18.79, 33.78)	57.69 (40.11, 76.44)	116.56 (81.04, 154.44)
	Both	33.79 (26.98, 41.22)	33.49 (26.74, 40.85)	114.28 (87.66, 143.21)	113.27 (86.88, 141.94)

40 to 44	Male	38.14 (29.13, 48.54)	73.52 (56.15, 93.56)	87.35 (65.85, 112.31)	168.37 (126.92, 216.49)
	Female	22.56 (16.45, 29.05)	45.33 (33.05, 58.36)	89.71 (64.59, 116.90)	180.26 (129.79,234.89)
	Both	60.70 (48.05, 74.35)	59.71 (47.28, 73.15)	177.06 (136.28, 221.11)	174.19 (134.07, 217.53)

45 to 49	Male	70.28 (52.48, 91.78)	113.65 (84.88, 148.42)	145.23 (106.41, 192.45)	234.87 (172.09, 311.24)
	Female	39.36 (28.81, 50.81)	66.12 (48.40, 85.35)	135.04 (96.92, 176.40)	226.83 (162.80, 296.31)
	Both	109.64 (86.10, 136.88)	90.34 (70.95, 112.78)	280.27 (212.93, 355.76)	230.93 (175.44, 293.13)

50 to 54	Male	124.92 (93.15, 162.43)	198.79 (148.23, 258.48)	238.60 (176.48, 312.06)	379.69 (280.83, 496.58)
	Female	69.48 (51.07, 89.46)	111.59 (82.03, 143.69)	198.32 (144.79, 257.11)	318.52 (232.55, 412.95)
	Both	194.40 (152.71, 241.11)	155.39 (122.06, 192.73)	436.92 (336.48, 549.15)	349.25 (268.96, 438.96)

55 to 59	Male	158.11 (118.62, 204.84)	331.86 (248.98, 429.95)	286.12 (211.65 ,373.97)	600.55 (444.24, 784.95)
	Female	81.77 (61.10, 104.84)	173.26 (129.46, 222.13)	198.91 (146.36, 257.25)	421.45 (310.09, 545.06)
	Both	239.88 (189.91, 296.72)	252.93 (200.24, 312.86)	485.03 (376.21, 605.99)	511.42 (396.68, 638.96)

60 to 64	Male	206.70 (157.72, 264.23)	523.76 (399.65, 669.56)	351.27 (267.99 ,449.95)	890.11 (679.09, 1140.15)
	Female	101.72 (77.37, 128.75)	260.21 (197.91, 329.36)	211.84 (159.82, 268.97)	541.91 (408.82, 688.04)
	Both	308.42 (248.13, 375.29)	392.61 (315.87, 477.74)	563.12 (448.63, 689.89)	716.84 (571.09, 878.21)

65 to 69	Male	268.42 (206.67, 337.09)	776.96 (598.23, 975.74)	432.51 (333.27, 546.17)	1251.93 (964.68, 1580.93)
	Female	135.91 (105.02, 168.98)	379.25 (293.04, 471.54)	245.47 (190.69, 305.73)	684.96 (532.10, 853.12)
	Both	404.33 (329.59, 484.54)	574.47 (468.27, 688.43)	677.97 (548.65, 820.74)	963.25 (779.51, 1166.09)

70 to 74	Male	277.08 (215.03, 345.62)	1187.74 (921.75, 1481.56)	412.65 (320.00, 516.11)	1768.92 (1371.76, 2212.42)
	Female	139.03 (108.08, 171.47)	566.81 (440.63, 699.08)	214.84 (166.62, 268.67)	875.92 (679.29, 1095.35)
	Both	416.10 (339.39, 494.39)	869.49 (709.19, 1033.09)	627.50 (511.19, 754.43)	1311.22 (1068.19, 1576.46)

75 to 79	Male	224.34 (177.25, 274.76)	1586.96 (1253.86, 1943.60)	299.22 (235.90, 369.68)	2116.59 (1668.73, 2615.06)
	Female	118.89 (92.58, 145.80)	756.80 (589.29, 928.11)	158.63 (123.11, 197.17)	1009.74 (783.65, 1255.09)
	Both	343.24 (283.28, 404.54)	1150.00 (949.13, 1355.38)	457.85 (376.02, 543.71)	1534.00 (1259.83, 1821.69)

80 plus	Male	283.24 (232.28, 333.00)	2405.36 (1972.62, 2827.94)	313.52 (258.21, 368.91)	2662.53 (2192.86, 3132.94)
	Female	194.01 (148.74, 233.90)	1049.41 (804.53,1265.21)	207.89 (159.34, 253.87)	1124.52 (861.91, 1373.23)
	Both	477.24 (399.09, 547.58)	1577.01 (1318.75, 1809.43)	521.41 (432.83, 602.13)	1722.96 (1430.24, 1989.70)

**Table 3:** All-age deaths and age-standardized mortality rates for different types of cancer and their percentage change by gender in China, 1990–2019.

Morphology	Gender	All-age deaths (95% UI), no. ∗ 10^3^(95% UI)			Age-standardized mortality rate (95% UI), per 100,000		
		1990	2019	Change, %	1990	2019	Change, %
All neoplasms	Male	868.85 (677.58, 1053.30)	1704.14 (1336.21,2112.01)	96.14	220.49 (175.99,264.00)	192.28(152.49,234.84)	-12.79
	Female	543.87 (426.76, 667.64)	936.05 (722.95,1154.37)	72.11	126.62 (100.21,154.35)	92.65 (71.67,113.95)	-26.82
	Both	1412.72 (1164.05,1661.26)	2640.20 (2174.68, 3131.22)	86.89	168.81 (140.27,196.93)	136.72(112.97,161.19)	-19.01

Tracheal, bronchus, and lung cancer	Male	177.93 (145.87,214.39)	523.19 (413.19,647.41)	194.04	46.33 (38.78,55.03)	58.1 (46.53,70.89)	25.40
	Female	78.4 (65.03,91.66)	233.98 (189.18,282.75)	198.44	18.63 (15.62,21.64)	22.86 (18.52,27.52)	22.71
	Both	256.33 (221.06,294.56)	757.17 (638.74,887.75)	195.39	31.18 (27.14,35.52)	38.7 (32.8,45.03)	24.12

Stomach cancer	Male	197.06 (161.82,232.16)	298.51 (238.47,363.78)	51.48	51.36 (42.96,59.94)	33.14 (26.67,39.89)	-35.48
	Female	108.41 (90.42,126.30)	123.03(99.95,150.64)	13.49	26.17 (21.94,30.37)	12.20 (9.93,14.92)	-53.38
	Both	305.47 (267.21,345.40)	421.54 (353.52,493.18)	38.00	37.73 (33.20,42.39)	21.72 (18.31,25.31)	-42.43

Colorectal cancer	Male	42.02 (34.9,49.40)	164.73 (132.24,202.32)	292.03	11.73 (9.95,13.59)	19.32 (15.8,23.15)	64.71
	Female	37.30 (31.31,43.32)	97.05 (78.38,117.17)	160.19	9.14 (7.73,10.58)	9.68 (7.82,11.68)	5.91
	Both	79.32 (69.66,89.29)	261.78 (224.4,303.32)	230.03	10.18 (9.03,11.37)	13.86 (11.92,16.01)	36.15

Esophageal cancer	Male	117.08 (73.11,141.72)	197.72 (151.48,246.01)	68.88	30.53 (19.80,36.44)	21.69 (16.61,26.66)	-28.96
	Female	59.52 (33.27,70.74)	59.6 (40.46,74.97)	0.13	14.69 (8.39,17.38)	5.92 (3.98,7.45)	-59.70
	Both	176.60 (112.85,206.04)	257.32 (202.78,309.03)	45.71	22.08 (14.14,25.53)	13.15 (10.27,15.68)	-40.44

Liver cancer	Male	164.91 (132.61,201.69)	139.03 (109.61,172.17)	-15.69	36.65 (29.77,44.3)	14.56 (11.63,17.86)	-60.27
	Female	67.54 (54.69,83.09)	48.67 (38.97,59.42)	-27.94	15.57 (12.70,19.04)	4.76 (3.81,5.80)	-69.43
	Both	232.45 (197.40,275.39)	187.7 (158.26,222.77)	-19.25	25.99 (22.29,30.55)	9.41 (7.95,11.13)	-63.79

Prostate cancer	Male	20.38 (15.82,24.68)	54.39 (42.9,71.31)	166.88	8.22 (6.62,10.28)	7.79 (6.22,9.94)	-5.23

Pancreatic cancer	Male	15.87 (12.94,19.23)	70.22 (55.62,86.99)	342.47	4.12 (3.42,4.91)	7.69 (6.15,9.41)	86.65
	Female	11.24 (9.46,13.07)	47.16 (38.00,57.33)	319.57	2.70 (2.28,3.13)	4.58 (3.70,5.56)	69.63
	Both	27.10 (23.6,30.84)	117.37 (99.86,136.45)	333.10	3.34 (2.93,3.76)	5.99 (5.12,6.93)	79.34

Cervical cancer	Female	26.42 (20.52,43.53)	53.44 (30.4,68.86)	102.27	5.85 (4.59,9.57)	5.13 (2.92,6.6)	-12.31

Breast cancer	Male	0.37 (0.30,0.45)	2.81 (2.15,3.53)	659.46	0.10 (0.08,0.12)	0.29 (0.22,0.36)	190.00
	Female	41.43 (34.15,49.15)	93.5 (74.51,115.42)	125.68	9.16 (7.61,10.82)	9.02 (7.19,11.10)	-1.53
	Both	41.80 (34.55,49.51)	96.31 (77.32,118.09)	130.41	4.74 (3.96,5.57)	4.85 (3.91,5.92)	2.32

Leukemia	Male	36.39 (25.18,43.85)	35.22 (26.33,44.01)	-3.22	6.88 (5.02,8.25)	4.42 (3.36,5.4)	-35.76
	Female	30.43 (24.36,37.13)	25.16 (19.08,31.27)	-17.32	5.83 (4.72,7.03)	3.00 (2.30,3.67)	-48.54
	Both	66.82 (54.35,77.29)	60.38 (50.21,70.99)	-9.64	6.28 (5.18,7.26)	3.67 (3.07,4.28)	-41.56

Brain and central nervous system cancer	Male	21.26 (14.5,31.89)	35.65 (22.01,47.44)	67.69	4.35 (3.02,6.46)	4.05 (2.53,5.33)	-6.90
	Female	16.71 (11.63,22.37)	27.88 (20.2,36.13)	66.85	3.44 (2.44,4.58)	3.01 (2.19,3.88)	-12.50
	Both	37.97 (29.10,50.21)	63.53 (47.79,76.95)	67.32	3.87 (3.04,5.10)	3.5 (2.62,4.21)	-9.56

Ovarian cancer	Female	8.04 (6.18,11.67)	29.09 (20.96,36.86)	261.82	1.76 (1.36,2.59)	2.77 (2.01,3.5)	57.39

Non-Hodgkin lymphoma	Male	9.45 (7.82,11.25)	29.12 (23.22,36.03)	208.15	2.16 (1.80,2.54)	3.19 (2.57,3.90)	47.69
	Female	6.98 (5.85,8.17)	15.19 (12.22,18.58)	117.62	1.53 (1.30,1.79)	1.55 (1.25,1.88)	1.31
	Both	16.44 (14.40,18.59)	44.31 (37.46,51.97)	169.53	1.82 (1.61,2.05)	2.32 (1.97,2.70)	27.47

Bladder cancer	Male	11.91 (9.94,13.77)	31.5 (25.60,38.33)	164.48	4.43 (3.79,5.04)	4.30 (3.56,5.12)	-2.93
	Female	5.38 (4.46,6.42)	8.59 (6.92,10.52)	59.67	1.45 (1.22,1.73)	0.88 (0.71,1.08)	-39.31
	Both	17.29 (15.09,19.45)	40.09 (33.98,47.18)	131.87	2.58 (2.26,2.90)	2.24 (1.91,2.61)	-13.18

Gallbladder and biliary tract cancer	Male	5.44 (4.31,9.16)	17.76 (12.88,22.42)	226.47	1.62 (1.33,2.59)	2.09 (1.53,2.6)	29.01
	Female	6.64 (5.14,11.48)	16.70 (10.30,21.30)	151.51	1.66 (1.29,2.84)	1.64 (1.01,2.09)	-1.20
	Both	12.08 (9.98,19.48)	34.46 (25.22,41.23)	185.26	1.61 (1.35,2.54)	1.82 (1.32,2.17)	13.04

Nasopharynx cancer	Male	17.43 (14.3,20.73)	21.35 (16.67,26.47)	22.49	3.86 (3.21,4.55)	2.20 (1.74,2.70)	-43.01
	Female	9.14 (7.28,11.02)	7.31 (5.7,9.04)	-20.02	2.01 (1.61,2.42)	0.72 (0.56,0.89)	-64.18
	Both	26.57 (22.77,30.28)	28.66 (23.78,34.07)	7.87	2.9 (2.51,3.3)	1.43 (1.19,1.69)	-50.69

Kidney cancer	Male	3.33 (2.73,4.07)	16.88 (13.18,20.84)	406.91	0.85 (0.7,1.01)	1.96 (1.55,2.38)	130.59
	Female	2.55 (2.19,2.94)	7.07 (5.68,8.62)	177.25	0.59 (0.50,0.67)	0.72 (0.59,0.87)	22.03
	Both	5.88 (5.14,6.69)	23.95 (19.77,28.48)	307.31	0.7 (0.61,0.79)	1.27 (1.05,1.49)	81.43

Uterine cancer	Female	10.60 (7.95,13.04)	12.22 (9.42,17.34)	15.28	2.38 (1.81,2.90)	1.17 (0.90,1.66)	-50.84

Lip and oral cavity cancer	Male	4.57 (3.72,5.45)	17.61 (14.09,21.90)	285.34	1.23 (1.02,1.44)	1.95 (1.59,2.40)	58.54
	Female	2.83 (2.36,3.33)	5.03 (4.06,6.12)	77.74	0.69 (0.57,0.80)	0.50 (0.41,0.61)	-27.54
	Both	7.40 (6.44,8.36)	22.64 (18.91,27.08)	205.95	0.92 (0.81,1.03)	1.16 (0.98,1.38)	26.09

Larynx cancer	Male	8.90 (7.24,10.49)	16.84 (13.50,20.73)	89.21	2.34 (1.94,2.72)	1.82 (1.47,2.21)	-22.22
	Female	2.59 (2.18,3.00)	3.42 (2.74,4.13)	32.05	0.62 (0.52,0.71)	0.34 (0.27,0.4)	-45.16
	Both	11.50 (9.85,13.24)	20.25 (16.76,24.23)	76.09	1.40 (1.21,1.60)	1.02 (0.85,1.21)	-27.14

Non-melanoma skin cancer	Male	2.69 (2.23,3.13)	7.98 (6.49,9.66)	196.65	0.88 (0.74,1)	1.05 (0.87,1.24)	19.32
	Female	2.29 (1.90,2.72)	7.50 (5.78,9.17)	227.51	0.59 (0.50,0.70)	0.77 (0.60,0.94)	30.51
	Both	4.98 (4.36,5.60)	15.48 (13.10,17.80)	210.84	0.70 (0.61,0.78)	0.88 (0.74,1.01)	25.71

Multiple myeloma	Male	2.60 (1.96,3.91)	7.71 (4.96,10.24)	196.54	0.65 (0.5,0.98)	0.83 (0.54,1.08)	27.69
	Female	2.74 (2.06,4.07)	5.71 (4.08,7.46)	108.39	0.64 (0.48,0.94)	0.55 (0.39,0.71)	-14.06
	Both	5.34 (4.35,7.53)	13.42 (10.06,16.5)	151.31	0.64 (0.53,0.89)	0.67 (0.50,0.82)	4.69

Thyroid cancer	Male	1.23 (1.00,1.52)	4.21 (3.18,5.24)	242.28	0.35 (0.29,0.43)	0.52 (0.40,0.64)	48.57
	Female	2.09 (1.71,2.68)	3.03 (2.40,3.76)	44.98	0.50 (0.41,0.63)	0.30 (0.24,0.38)	-40.00
	Both	3.32 (2.86,4.13)	7.24 (6.01,8.48)	118.07	0.42 (0.37,0.53)	0.39 (0.32,0.45)	-7.14

Other pharynx cancer	Male	2.26 (1.86,2.67)	4.60 (3.55,5.74)	103.54	0.58 (0.49,0.69)	0.49 (0.38,0.6)	-15.52
	Female	0.73 (0.60,0.87)	0.99 (0.79,1.21)	35.62	0.17 (0.14,0.20)	0.10 (0.08,0.12)	-41.18
	Both	2.99 (2.57,3.44)	5.59 (4.53,6.73)	86.96	0.36 (0.31,0.41)	0.28 (0.23,0.33)	-22.22

Malignant skin melanoma	Male	1.45 (0.88,2.02)	2.5 (1.45,3.47)	72.41	0.34 (0.22,0.48)	0.29 (0.17,0.40)	-14.71
	Female	1.32 (0.81,2.19)	2.66 (1.42,3.64)	101.52	0.31 (0.20,0.51)	0.27 (0.15,0.37)	-12.90
	Both	2.76 (1.90,4.06)	5.16 (3.28,6.50)	86.96	0.33 (0.23,0.48)	0.28 (0.18,0.35)	-15.15

Testicular cancer	Male	0.78 (0.65,0.91)	1.21 (0.96,1.47)	55.13	0.16 (0.13,0.18)	0.16 (0.13,0.19)	0.00

Hodgkin lymphoma	Male	3.03 (1.45,4.01)	1.76 (1.18,2.35)	-41.91	0.65 (0.31,0.85)	0.20 (0.13,0.26)	-69.23
	Female	1.92 (0.83,2.68)	0.95 (0.64,1.23)	-50.52	0.40 (0.18,0.55)	0.10 (0.07,0.13)	-75.00
	Both	4.95 (2.57,6.47)	2.71 (2.00,3.29)	-45.25	0.52 (0.27,0.67)	0.15 (0.11,0.18)	-71.15

Mesothelioma	Male	0.51 (0.40,0.74)	1.65 (1.29,2.14)	223.53	0.12 (0.10,0.18)	0.18 (0.14,0.23)	50.00
	Female	0.62 (0.41,1.02)	1.12 (0.72,1.44)	80.65	0.14 (0.10,0.23)	0.11 (0.07,0.14)	-21.43
	Both	1.13 (0.86,1.59)	2.77 (2.28,3.32)	145.13	0.13 (0.10,0.19)	0.14 (0.12,0.17)	7.69

**Table 4:** All-age disability-adjusted life-years (DALYs) and age-standardized DALY rates for different types of cancer and their percentage change by gender in China, 1990–2019.

Morphology	Gender	All-age DALYs, no. ∗ 10^3^(95% UI)			Age-standardized DALY Rate (95% UI), per 100,000		
		1990	2019	Change, %	1990	2019	Change, %
All neoplasms	Male	26665.90 (20491.53, 32634.11)	42241.06 (32906.13, 52726.38)	58.41	5642.34 (4386.93,6859.92)	4375.35 (3430.65,5424.80)	-22.46
	Female	16502.20 (12752.07, 20516.94)	23028.20 (17744.80,28622.55)	39.55	3456.54 (2687.74,4276.93)	2301.10 (1773.10,2853.61)	-33.43
	Both	43168.10 (35243.76,51219.97)	65269.26 (53487.48,77903.09)	51.20	4516.45 (3703.94,5337.17)	3288.22 (2704.53,3910.50)	-27.19

Tracheal, bronchus, and lung cancer	Male	4864.23 (3954.89,5914.77)	11967.77 (9374.37,14952.33)	146.04	1084.53 (891.58,1310.23)	1203.78 (950.19,1495.1)	11.00
	Female	2096.64 (1730.33,2468.93)	5160.82 (4138.83,6353.14)	146.15	456.89 (377.41,535.53)	492.17 (393.74,604.27)	7.72
	Both	6960.87 (5966.96,8039.13)	17128.58 (14340.49,20231.34)	146.07	760.68 (654.28,875.37)	831.27 (699.11,979.99)	9.28

Stomach cancer	Male	5409.61 (4428.71,6387.35)	7135.99 (5633.55,8786.15)	31.91	1205.79 (992.78,1421.38)	718.79 (571.96,877.30)	-40.39
	Female	2839.18 (2341.70,3338.41)	2689.00 (2162.24,3333.93)	-5.29	619.82 (513.84,726.15)	260.97 (209.75,323.21)	-57.90
	Both	8248.79 (7173.64,9366.34)	9824.99 (8191.72,11632.86)	19.11	905.54 (791.75,1024.49)	481.15 (403.20,567.36)	-46.87

Colorectal cancer	Male	1237.93 (1024.37,1469.8)	4168.63 (3354.03,5154.83)	236.74	272.77 (228.96,321.10)	434.5 (353.42,531.60)	59.29
	Female	1033.38 (860.91,1210.10)	2226.29 (1811.95,2708.17)	115.44	223.02 (186.75,260.80)	217.28 (176.73,264.24)	-2.57
	Both	2271.31 (1988.09,2566.70)	6394.92 (5462.29,7408.70)	181.55	245.60 (215.43,276.40)	320.57 (275.4,370.70)	30.53

Esophageal cancer	Male	3127.52 (1947.69,3810.76)	4622.3 (3552.36,5810.37)	47.79	707.12 (443.86,855.35)	458.55 (354.39,572.18)	-35.15
	Female	1366.55 (742.99,1637.54)	1137.70 (809.67,1428.72)	-16.75	311.63 (172.06,371.98)	108.46 (76.74,135.97)	-65.20
	Both	4494.07 (2848.08,5295.22)	5760.00 (4581.77,6999.57)	28.17	506.98 (321.63,593.47)	277.50 (221.69,335.87)	-45.26

Liver cancer	Male	5590.40 (4500.90,6841.76)	4145.80 (3241.13,5167.31)	-25.84	1102.84 (889.18,1343.26)	414.90 (326.97,514.24)	-62.38
	Female	1987.37 (1593.34,2470.03)	1179.67 (936.01,1453.06)	-40.64	420.06 (338.28,520.01)	115.85 (92.14,142.16)	-72.42
	Both	7577.77 (6419.83,8981.02)	5325.46 (4425.69,6374.59)	-29.72	769.11 (653.21,913.07)	264.31 (220.69,315.14)	-65.63

Leukemia	Male	2130.99 (1362.49,2578.72)	1365.39 (1030.96,1668.32)	-35.93	352.83 (229.15,429.38)	190.81 (144.9,235.81)	-45.92
	Female	1762.75 (1317.40,2177.36)	943.92 (729.07,1151.66)	-46.45	315.55 (238.95,387.64)	135.97 (106.51,162.94)	-56.91
	Both	3893.74 (3068.11,4551.14)	2309.31 (1924.58,2691.12)	-40.69	333.77 (263.69,390.45)	163.82 (136.6,189.48)	-50.92

Cervical cancer	Female	855.36 (654.48,1432.99)	1622.24 (892.58,2090.86)	89.66	176.4 (135.68,294.69)	157.50 (86.90,202.91)	-10.71

Breast cancer	Male	11.62 (9.46,14.00)	80.21 (61.44,100.88)	590.28	2.45 (2.00,2.93)	7.73 (5.98,9.62)	215.51
	Female	1423.49 (1173.66,1696.00)	2877.24 (2323.69,3513.54)	102.13	294.04 (243.51,349.12)	277.98 (224.35,339.93)	-5.46
	Both	1435.10 (1184.14,1708.68)	2957.45 (2408.51,3590.17)	106.08	145.67 (121.08,172.57)	144.15 (117.26,174.99)	-1.04

Pancreatic cancer	Male	455.55 (367.84,555.91)	1760.52 (1381.02,2203.79)	286.46	99.25 (81.00,120.14)	176.41 (139.31,219.18)	77.74
	Female	293.87 (243.00,345.65)	1044.66 (837.73,1279.10)	255.48	64.48 (53.69,75.61)	98.93 (79.22,121.02)	53.43
	Both	749.41 (644.18,863.72)	2805.18 (2368.77,3276.55)	274.32	81.48 (70.48,93.24)	136.57 (115.59,158.93)	67.61

Brain and central nervous system cancer	Male	1005.83 (655.96,1568.96)	1178.28 (743.32,1566.66)	17.15	178.20 (117.52,276.17)	143.02 (89.74,189)	-19.74
	Female	763.83 (493.38,1042.82)	875.14 (649.70,1156.06)	14.57	144.01 (93.80,195.96)	109.48 (80.78,144.78)	-23.98
	Both	1769.66 (1286.81,2432.38)	2053.42 (1584.34,2524.97)	16.03	161.29 (118.00,220.25)	126.24 (96.01,154.80)	-21.73

Prostate cancer	Male	403.11 (306.18,488.11)	1002.59 (794.01,1322.63)	148.71	125.93 (99.53,151.84)	118.94 (95.05,154.14)	-5.55

Ovarian cancer	Female	275.06 (212.26,377.97)	835.06 (612.56,1063.25)	203.59	55.57 (42.86,77.80)	80.52 (59.44,102.57)	44.90

Non-Hodgkin lymphoma	Male	379.06 (316.14,449.30)	878.86 (702.20,1085.95)	131.85	70.67 (59.36,83.47)	95.58 (77.16,116.59)	35.25
	Female	271.02 (228.59,317.56)	427.38 (345.89,521.43)	57.69	52.27 (44.2,61.23)	46.95 (38.28,56.63)	-10.18
	Both	650.08 (572.42,734.67)	1306.25 (1103.33,1521.35)	100.94	61.41 (54.34,69.17)	71.00 (60.64,81.81)	15.62

Nasopharynx cancer	Male	624.47 (514.94,742.33)	692.83 (547.16,856.24)	10.95	120.19 (99.50,142.34)	69.51 (55.30,85.49)	-42.17
	Female	311.06 (248.27,375.31)	219.28 (172.23,273.09)	-29.51	62.44 (49.82,75.26)	21.97 (17.34,27.27)	-64.81
	Both	935.53 (799.02,1072.20)	912.11 (760.83,1081.93)	-2.50	91.67 (78.49,104.64)	45.60 (38.3,53.80)	-50.26

Bladder cancer	Male	280.05 (232.44,326.32)	651.10 (528.55,791.98)	132.49	76.38 (64.43,87.29)	73.49 (60.47,88.56)	-3.78
	Female	125.51 (103.00,150.03)	165.02 (132.47,202.22)	31.48	28.99 (23.89,34.61)	16.22 (13.05,19.85)	-44.05
	Both	405.56 (353.23,459.12)	816.12 (691.35,967.38)	101.23	49.41 (43.22,55.60)	41.88 (35.59,49.34)	-15.24

Gallbladder and biliary tract cancer	Male	146.74 (115.52,241.03)	410.61 (293.21,522.30)	179.82	34.26 (27.38,56.37)	42.60 (30.64,53.79)	24.34
	Female	162.28 (123.28,279.48)	352.97 (220.46,453.95)	117.51	36.46 (27.95,62.67)	33.57 (20.96,43.23)	-7.93
	Both	309.02 (254.14,486.15)	763.58 (566.76,920.49)	147.10	35.18 (29.16,56.02)	37.71 (27.91,45.35)	7.19

Uterine cancer	Female	332.05 (238.02,411.58)	364.28 (286.56,503.57)	9.71	69.61 (50.73,85.85)	34.93 (27.27,47.77)	-49.82

Kidney cancer	Male	121.56 (100.81,146.97)	462.87 (363.08,571.56)	280.77	24.44 (20.39,29.50)	49.65 (39.57,60.57)	103.15
	Female	94.21 (81.14,107.59)	179.93 (147.91,216.68)	90.99	19.04 (16.49,21.63)	20.07 (16.75,23.75)	5.41
	Both	215.76 (190.36,243.56)	642.8 (533.66,763.98)	197.92	21.59 (19.04,24.40)	34.28 (28.95,40.16)	58.78

Lip and oral cavity cancer	Male	138.59 (112.35,166.48)	454.78 (360.26,571.48)	228.15	29.96 (24.49,35.71)	45.75 (36.55,56.94)	52.70
	Female	84.05 (69.03,99.39)	121.02 (97.7,147.27)	43.99	17.75 (14.66,21.05)	11.94 (9.65,14.51)	-32.73
	Both	222.65 (192.53,252.40)	575.81 (479.52,690.74)	158.62	23.63 (20.48,26.66)	28.27 (23.59,33.71)	19.64

Larynx cancer	Male	249.57 (201.04,296.26)	421.57 (337.84,519.58)	68.92	55.71 (45.4,65.32)	41.54 (33.47,51.01)	-25.44
	Female	68.15 (56.64,79.70)	76.36 (61.43,92.81)	12.05	14.87 (12.41,17.29)	7.34 (5.92,8.90)	-50.64
	Both	317.72 (268.27,367.83)	497.93 (411.84,600.18)	56.72	34.78 (29.53,40.08)	23.85 (19.81,28.62)	-31.43

Multiple myeloma	Male	75.98 (57.16,114.32)	203.66 (130.78,268.80)	168.04	16.32 (12.36,24.48)	20.57 (13.12,26.99)	26.04
	Female	72.50 (54.07,109.05)	143.79 (99.65,187.87)	98.33	15.77 (11.8,23.42)	13.79 (9.45,17.82)	-12.56
	Both	148.48 (120.36,211.47)	347.45 (253.91,424.33)	134.00	16.03 (13.02,22.62)	17.05 (12.31,20.77)	6.36

Non-melanoma skin cancer	Male	69.06 (56.89,81.03)	169.95 (136.49,208.25)	146.09	16.78 (13.93,19.51)	18.90 (15.39,22.89)	12.63
	Female	59.94 (48.96,72.39)	154.42 (116.41,191.68)	157.62	13.21 (10.81,15.87)	15.23 (11.52,18.82)	15.29
	Both	128.99 (112.67,146.36)	324.38 (272.52,376.46)	151.48	14.60 (12.82,16.49)	16.76 (14.18,19.38)	14.79

Thyroid cancer	Male	38.44 (31.36,46.79)	107.11 (81.45,133.26)	178.64	8.36 (6.89,10.21)	11.64 (8.91,14.29)	39.23
	Female	65.05 (52.36,80.81)	80.21 (64.74,99.49)	23.31	13.48 (10.98,16.66)	8.11 (6.55,10.07)	-39.84
	Both	103.49 (87.96,124.72)	187.32 (156.24,219.11)	81.00	10.87 (9.33,13.21)	9.7 (8.11,11.27)	-10.76

Malignant skin melanoma	Male	53.37 (32.04,74.11)	79.89 (45.35,108.93)	49.69	10.25 (6.22,14.22)	8.57 (4.96,11.69)	-16.39
	Female	42.42 (25,70.61)	70.23 (37.33,96.33)	65.56	8.62 (5.18,14.29)	7.32 (3.94,9.99)	-15.08
	Both	95.79 (63.30,137.80)	150.12 (93.91,189.54)	56.72	9.48 (6.41,13.87)	7.96 (5.06,9.99)	-16.03

Other pharynx cancer	Male	68.13 (55.59,81.27)	121.75 (92.99,152.68)	78.70	14.66 (12.10,17.33)	11.83 (9.13,14.79)	-19.30
	Female	20.48 (16.67,24.36)	23.73 (18.84,29.23)	15.87	4.38 (3.59,5.21)	2.30 (1.82,2.82)	-47.49
	Both	88.61 (75.24,102.57)	145.47 (117.12,176.32)	64.17	9.42 (8.06,10.85)	6.95 (5.61,8.40)	-26.22

Testicular cancer	Male	39.45 (33.11,45.47)	51.15 (42.02,61.19)	29.66	6.43 (5.42,7.41)	6.70 (5.52,7.90)	4.20

Hodgkin lymphoma	Male	127.11 (60.21,167.15)	56.81 (39.45,75.09)	-55.31	22.73 (10.79,29.93)	6.50 (4.61,8.58)	-71.40
	Female	77.23 (31.88,110.31)	29.36 (20.85,38.34)	-61.98	14.31 (5.91,20.26)	3.44 (2.49,4.52)	-75.96
	Both	204.34 (103.13,269.11)	86.17 (65.53,105.33)	-57.83	18.56 (9.44,24.32)	4.95 (3.85,6.08)	-73.33

Mesothelioma	Male	17.53 (13.46,25.13)	50.64 (39.12,65.81)	188.88	3.49 (2.71,5.05)	5.09 (3.94,6.55)	45.85
	Female	18.79 (11.73,30.96)	28.48 (18.31,37.08)	51.57	3.87 (2.49,6.34)	2.81 (1.81,3.66)	-27.39
	Both	36.32 (27.23,51.58)	79.12 (65.07,94.57)	117.84	3.68 (2.80,5.23)	3.94 (3.24,4.7)	7.07

**Table 5:** Trends in ASRs of incidence, mortality, and DALY for selected cancers by sex in China, 1990-2019.

Morphology	Gender	AAPC 1990-2019 (95% CI)		
		ASIR	ASMR	ASRs of DALY
All neoplasms	Male	0.88 (0.81,0.96)	-0.46 (-0.58,-0.34)	-0.97 (-1.07,-0.87)
	Female	0.51 (0.40,0.61)	-1.24 (-1.40,-1.08)	-1.66 (-1.80,-1.51)
	Both	0.71 (0.62,0.79)	-0.80 (-0.94,-0.66)	-1.26 (-1.38,-1.14)

Tracheal, bronchus, and lung cancer	Male	1.49 (1.31,1.66)	-0.43 (-0.64,-0.21)	0.60 (0.43,0.77)
	Female	1.08 (0.90,1.26)	0.67 (0.47,0.88)	0.11 (-0.07,0.30)
	Both	1.33 (1.16,1.50)	0.94 (0.74,1.14)	0.42 (0.25,0.59)

Stomach cancer	Male	0.14 (-0.21,0.50)	-1.17 (-1.58,-0.77)	-1.47 (-1.89,-1.05)
	Female	-1.60 (-1.95,-1.25)	-2.59 (-3.00,-2.17)	-3.02 (-3.41,-2.63)
	Both	-0.42 (-0.77,-0.06)	-1.68 (-2.09,-1.27)	-1.98 (-2.4,-1.57)

Colorectal cancer	Male	4.46 (4.11,4.82)	2.21 (1.92,2.50)	2.12 (1.81,2.43)
	Female	2.50 (2.26,2.74)	0.32 (0.11,0.52)	-0.03 (-0.19,0.13)
	Both	3.66 (3.36,3.97)	1.39 (1.13,1.64)	1.24 (1.00,1.49)

Breast cancer	Male	8.74 (7.59,9.89)	6.21 (5.21,7.22)	6.59 (5.51,7.67)
	Female	2.70 (2.60,2.79)	-0.14 (-0.21,-0.06)	-0.35 (-0.43,-0.26)
	Both	2.84 (2.74,2.95)	0.06 (0,0.13)	-0.13 (-0.19,-0.06)

Prostate cancer	Male	2.54 (2.44,2.64)	-0.22 (-0.27,-0.17)	-0.24 (-0.28,-0.20)
Non-melanoma skin cancer	Male	3.95 (3.46,4.45)	1.27 (0.99,1.55)	0.99 (0.73,1.25)
	Female	3.77 (3.30,4.25)	1.67 (1.32,2.03)	1.16 (0.86,1.46)
	Both	3.87 (3.39,4.35)	1.48 (1.17,1.79)	1.09 (0.82,1.37)

Esophageal cancer	Male	-0.96 (-1.37,-0.55)	-1.21 (-1.64,-0.78)	-1.56 (-2.03,-1.10)
	Female	-2.85 (-3.45,-2.26)	-3.53 (-4.14,-2.91)	-4.11 (-4.77,-3.43)
	Both	-1.58 (-2.05,-1.11)	-1.96 (-2.44,-1.47)	-2.27 (-2.78,-1.75)

Cervical cancer	Female	1.61 (1.35,1.88)	0.09 (-0.18,0.36)	0.16 (-0.09,0.41)
Leukemia	Male	-0.61 (-0.74,-0.48)	-1.60 (-1.66,-1.53)	-2.33 (-2.43,-2.24)
	Female	-1.56 (-1.84,-1.27)	-2.62 (-2.82, -2.41)	-3.37 (-3.67,-3.08)
	Both	-1.06 (-1.25,-0.86)	-2.05 (-2.17,-1.93)	-2.79 (-2.97,-2.61)

Liver cancer	Male	-4.40 (-5.27,-3.53)	-4.81 (-5.66,-3.94)	-5.05 (-5.94,-4.15)
	Female	-5.16(-5.86,-4.45)	-5.42 (-6.12,-4.71)	-5.87 (-6.62,-5.1)
	Both	-4.68(-5.50,-3.84)	-5.06 (-5.88,-4.24)	-5.31 (-6.17,-4.44)

Uterine cancer	Female	1.26 (0.58,1.94)	-2.27 (-2.88,-1.66)	-2.21 (-2.81,-1.60)
Pancreatic cancer	Male	2.64 (2.43,2.85)	2.55 (2.34,2.75)	2.39 (2.17,2.62)
	Female	1.88 (1.70,2.07)	1.85 (1.66,2.04)	1.45 (1.29,1.61)
	Both	2.32 (2.12,2.51)	2.25 (2.05,2.45)	2.02(1.83,2.21)

Brain and central nervous system cancer	Male	0.50 (0.45,0.55)	-0.30 (-0.39,-0.21)	-0.94 (-1.04,-0.84)
	Female	1.07 (0.97,1.18)	-0.70 (-0.83,-0.57)	-1.37 (-1.58,-1.17)
	Both	0.76 (0.68,0.83)	-0.49 (-0.59,-0.38)	-1.13 (-1.28,-0.99)

Nasopharynx cancer	Male	2.68 (2.28,3.08)	-2.14 (-2.25,-2.03)	-2.10 (-2.22,-1.98)
	Female	0.68 (0.44,0.91)	-3.96 (-4.15,-3.77)	-4.12 (-4.37,-3.87)
	Both	2.07 (1.72,2.41)	-2.69 (-2.82,-2.57)	-2.71 (-2.87,-2.56)

Bladder cancer	Male	2.14 (2.02,2.26)	-0.07 (-0.12,-0.01)	-0.07 (-0.12,-0.02)
	Female	-0.10 (-0.19,-0.01)	-1.89 (-1.97,-1.81)	-2.20 (-2.29,-2.12)
	Both	1.74 (1.63,1.85)	-0.53 (-0.58,-0.49)	-0.58 (-0.64,-0.53)

Non-Hodgkin lymphoma	Male	4.58 (4.17,5.00)	2.06 (1.75,2.36)	1.66 (1.37,1.95)
	Female	2.38 (2.15,2.60)	-0.13 (-0.32,0.05)	-0.75(-1.03,-0.46)
	Both	3.72 (3.40,4.04)	1.20 (0.98,1.43)	0.74 (0.51,0.97)

Ovarian cancer	Female	1.88 (1.79,1.98)	1.52 (1.42,1.61)	1.15 (1.04,1.25)

Kidney cancer	Male	5.46 (4.88,6.05)	3.82 (3.30,4.34)	3.33 (2.82,3.85)
	Female	2.80 (2.38,3.23)	1.17 (0.80,1.53)	0.55 (0.19,0.90)
	Both	4.46 (3.95,4.98)	2.79 (2.33,3.26)	2.28 (1.84,2.73)

Testicular cancer	Male	5.38 (5.22,5.54)	-0.43 (-0.64,-0.21)	-0.25 (-0.47,-0.04)

Lip and oral cavity cancer	Male	3.57 (3.13,4.00)	2.56 (2.17,2.96)	2.41 (2.02,2.81)
	Female	-0.10 (-0.19,-0.02)	-1.17 (-1.23,-1.11)	-1.57 (-1.66,-1.48)
	Both	2.33 (2.02,2.64)	1.44 (1.14,1.73)	1.24 (0.96,1.52)

Larynx cancer	Male	1.59 (1.37,1.81)	-0.74 (-0.86,-0.63)	-0.94 (-1.04,-0.84)
	Female	-0.02 (-0.32,0.28)	-1.83 (-2.05,-1.62)	-2.23 (-2.41,-2.06)
	Both	1.32 (1.11,1.54)	-0.96 (-1.07,-0.86)	-1.21 (-1.30,-1.12)

Thyroid cancer	Male	4.99 (4.59,5.39)	2.20 (1.86,2.54)	1.84 (1.56,2.13)
	Female	1.57 (1.45,1.68)	-1.79 (-1.86,-1.71)	-2.00 (-2.11,-1.90)
	Both	2.73 (2.58,2.88)	0.06 (-0.09,0.21)	-0.20 (-0.31,-0.10)

Gallbladder and biliary tract cancer	Male	2.10 (1.66,2.55)	1.68 (1.25,2.11)	1.55 (1.1,1.99)
	Female	1.07 (0.60,1.55)	0.58 (0.12,1.04)	0.30 (-0.14,0.75)
	Both	1.56 (1.1,2.02)	1.10 (0.65,1.54)	0.93 (0.49,1.37)

Multiple myeloma	Male	1.85 (1.76,1.95)	0.93 (0.85,1.00)	0.87 (0.80,0.94)
	Female	0.10 (0.03,0.18)	-0.64 (-0.71,-0.56)	-0.64 (-0.73,-0.54)
	Both	1.04 (0.96,1.12)	0.17 (0.10,0.23)	0.17 (0.10,0.25)

Malignant skin melanoma	Male	3.23 (2.91,3.55)	-0.58 (-0.67,-0.48)	-0.55 (-0.67,-0.43)
	Female	3.49 (3.18,3.80)	-0.32 (-0.45,-0.18)	-0.55 (-0.70,-0.41)
	Both	3.35 (3.03,3.67)	-0.45 (-0.56,-0.33)	-0.56 (-0.69,-0.43)

Hodgkin lymphoma	Male	-0.11 (-0.54,0.32)	-4.39 (-4.63,-4.14)	-4.69 (-4.97,-4.40)
	Female	-1.13 (-1.57,-0.69)	-5.34 (-5.67,-5.01)	-5.67 (-6.09,-5.26)
	Both	-0.54 (-0.96,-0.13)	-4.77 (-5.04,-4.50)	-5.07 (-5.4,-4.75)

Other pharynx cancer	Male	0.92 (0.59,1.24)	-0.80 (-1.04,-0.56)	-0.91 (-1.12,-0.69)
	Female	0.87 (0.61,1.13)	-2.01 (-2.12,-1.90)	-2.38 (-2.47,-2.30)
	Both	0.96 (0.67,1.24)	-1.01 (-1.19,-0.83)	-1.21 (-1.39,-1.04)

Mesothelioma	Male	2.26 (1.78,2.75)	2.32 (1.83,2.82)	2.42(1.92,2.92)
	Female	-0.66 (-0.83,-0.48)	-0.59 (-0.78,-0.41)	-0.89(-1.03,-0.75)
	Both	0.91 (0.57,1.26)	0.93 (0.59,1.28)	0.95(0.61,1.30)

## Data Availability

Data used for this publication are available on the website of the Institute for Health Metrics and Evaluation (available at http://ghdx.healthdata.org/gbd-results-tool) and can be browsed or downloaded with a free access.
